# A Moderate Daily Dose of Resveratrol Mitigates Muscle Deconditioning in a Martian Gravity Analog

**DOI:** 10.3389/fphys.2019.00899

**Published:** 2019-07-18

**Authors:** Marie Mortreux, Daniela Riveros, Mary L. Bouxsein, Seward B. Rutkove

**Affiliations:** ^1^Department of Neurology, Beth Israel Deaconess Medical Center, Harvard Medical School, Boston, MA, United States; ^2^Center for Advanced Orthopaedic Studies, Beth Israel Deaconess Medical Center, Harvard Medical School, Boston, MA, United States

**Keywords:** partial gravity, resveratrol, rats, Mars, muscle function

## Abstract

While there is a relatively good understanding of the effects of microgravity on human physiology based on five decades of experience, the physiological consequences of partial gravity remain far less well understood. Until recently, no model had been able to replicate partial gravity such as that experienced on Mars (0.38 g), which would be critical to help sustain long-term missions and ensure a safe return to Earth. Recent development of two partial weight bearing (PWB) models, one in mice and one in rats, now allows for quadrupedal partial unloading that mimics Martian gravity. Resveratrol (RSV), a polyphenol most commonly found in grapes and blueberries, has been extensively investigated for its health benefits, including its anti-inflammatory, anti-oxidative, and anti-diabetic effects. In the context of mechanical unloading, RSV has also been shown to preserve bone and muscle mass. However, there is a lack of research regarding its effect on the musculoskeletal system in partial gravity. We hypothesized that a moderate daily dose of RSV (150 mg/kg/day) would help mitigate muscle deconditioning in a Mars gravity analog. Indeed, our results demonstrate that RSV treatment during partial unloading significantly preserves muscle function (e.g., the average change in grip force after 14 days of PWB40 was of −6.18, and +10.92% when RSV was administered) and mitigates muscle atrophy (e.g., RSV supplementation led to an increase of 21.6% in soleus weight for the unloaded animals). This work suggests the potential of a nutraceutical approach to reduce musculoskeletal deconditioning on a long-term mission to Mars.

## Introduction

After decades of manned low earth orbit missions, space agencies are now targeting other planets for human exploration. In the upcoming years, NASA plans to send astronauts to the Moon and to Mars (National Aeronautics and Space Administration [NASA], 2018), both of which display a significantly lower gravity than Earth (0.16 and 0.38 g, respectively). While there is an extensive literature reviewing the impact of microgravity (real or simulated) on the muscular system (Fitts et al., 2000; Morey-Holton et al., 2005; Baldwin et al., 2013; Chowdhury et al., 2013; Ohira et al., 2015; Radugina et al., 2018), relatively little is known about the effects of partial gravity. Thus, mitigating strategies will be needed to prevent muscle deconditioning and allow astronauts to safely perform tasks upon arrival on Mars, especially after being reintroduced to gravity, even if at a reduced level.

Among potential medicinal interventions to prevent muscle deterioration, nutraceuticals are especially appealing since they are naturally occurring compounds that are present in the diet. Moreover, astronauts en route to Mars will not have the opportunity to exercise with the same devices currently deployed on the ISS (Petersen et al., 2016). Of potential candidates, resveratrol (RSV), a polyphenol, commonly found in grape skin, red wine, and blueberries and used as a dietary supplement, has been widely investigated (Tran et al., 2017; Yonamine et al., 2017; Zhang et al., 2017, 2018; Zhu et al., 2017; Franck et al., 2018), and declared safe for animals and humans (Williams et al., 2009). RSV has already been studied for its osteoprotective effects (Shen et al., 2012; Durbin et al., 2014; Ornstrup et al., 2014), and described as a physical exercise mimetic to prevent wasting disorders during hindlimb unloading (Momken et al., 2011). However, its mitigating effect has never been studied in a partial weight-bearing (PWB) model.

In this study, we hypothesized that daily supplementation with a moderate dose of RSV would mitigate muscle impairment in a ground-based partial gravity analog that mimics a Martian environment (0.4 g, PWB40). If correct, our results would offer proof of concept for such intervention with subsequent efforts devoted to unraveling potential underlying mechanisms of action.

## Materials and Methods

### Animals and Supplementation

Twenty-four male Wistar rats (Charles River Laboratories Inc., Wilmington, MA, United States) weighing 425 ± 6.73 g (14 weeks of age) were obtained and housed in a temperature-controlled (22 ± 2°C) room with a 12:12-hour light-dark cycle. Water and rat chow were provided *ad libitum* and daily food intake was monitored. Animals were exposed to normal loading (PWB100) or Martian gravity (40% of normal loading: PWB40) for 14 days. Detailed description about the control group, the housing environment and the suspension apparatus can be found in our previous articles describing this model (Mortreux et al., 2018, 2019). Half of the animals in each group received resveratrol daily (RSV, 150 mg/kg/day, Transreveratrol HPLC Purified, megaresveratrol.net, Candlewood Stars, Inc, Danbury, CT, United States) starting at baseline (day 0). RSV was prepared daily by mixing the powder with 10% sucrose water into a 150 mg/ml solution that was administered orally using a 1 mL syringe. Animals were weighed weekly, free of their suspension apparatus. All experimental protocols were approved by the Beth Israel Deaconess Medical Center Institutional Animal Care and Use Committee.

### Longitudinal Limb Muscle Assessment

Weekly, calf circumference was measured at the left tibia mid-shaft. Animals were anesthetized with isoflurane and placed in a prone position with their left hind limb taped at a 45-degree angle. Fur was removed using a hair clipper (Braintree Scientific, Braintree, MA, United States) and suture thread was used to obtain three circumference measurements and the average was recorded.

Using a 50 N grip force meter (Chatillon, Largo, FL, United States), both front and rear paw grip force were assessed weekly by performing three gentle pulls until the animals released their grip, allowing them to rest for 30 s between trials. The peak force of each trial was recorded and the three trials averaged.

### Terminal Muscle Assessment

After 14 days, animals were sacrificed and the triceps surae (e.g., soleus and gastrocnemius muscles) was harvested and muscle wet mass was measured using a precision balance (Fisher Scientific, Pittsburgh, PA, United States). Muscles were fixed in 10% formalin for 48 h and embedded in paraffin. Immunohistochemical analysis was performed using anti-collagen VI (ab6588, Abcam, Cambridge, MA, United States) and anti-slow skeletal myosin heavy chain (ab11083, Abcam, Cambridge, MA, United States) antibodies. Images were obtained at 20× using an epifluorescence microscope (Zeiss Axio Imager M1) and a minimum of 100 myofibers were analyzed for each muscle with FIJI (ImageJ, NIH) using the muscle morphometry plug-in (Anthony Sinadinos using Eclipse IDE) to calculate the average myofiber cross sectional area (CSA) with the experimenter blinded for treatment and gravity level.

### Statistical Analyses

Longitudinal measurements were analyzed using repeated measures 2-way ANOVA followed by Tukey’s multiple comparisons test. Terminal measurements were analyzed using 1-way ANOVA followed by Tukey’s *post hoc* test. All results were considered significant when *p* < 0.05 and performed using GraphPad Prism 7.

## Results

**FIGURE 1:**
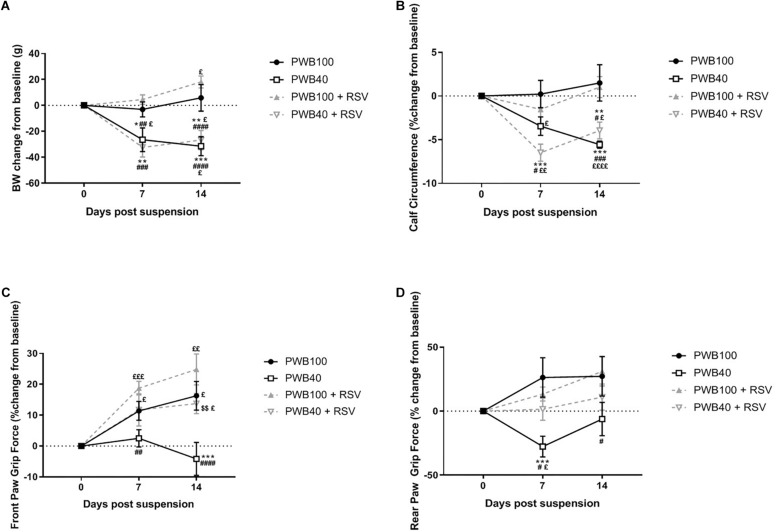
Weekly changes in body weight **(A)**, calf circumference **(B)**, front paw **(C)** and rear paw **(D)** grip force compared to pre-suspension values (day 0). Data are presented as mean ± SEM; results of the *post hoc* tests following the ANOVA are indicated. *N* = 6 per group. ^*^p < 0.05, ^∗∗^*p* < 0.01, ^∗∗∗^*p* < 0.001, and ^*⁣*⁣**^p < 0.0001 vs. PWB100, respectively. ^#^*p* < 0.05, ^##^*p* < 0.01, ^###^*p* < 0.001, and ^#⁢#⁢#⁢#^*p* < 0.0001 vs. PWB100 + RSV, respectively. ^$$^*p* < 0.01 vs. PWB40. ^£^*p* < 0.05, ^£⁢£^*p* < 0.01, ^£⁢£⁢£^*p* < 0.001, and ^£⁢£⁢£⁢£^*p* < 0.0001 vs. baseline values (day 0), respectively.

Resveratrol supplementation did not influence body weight (Figure 1A) in any group as compared to un-supplemented animals. Calf circumference was not impacted by the RSV supplementation and animals from both PWB40 and PWB40 + RSV groups displayed a significant reduction over time compared to either their baseline values or to the two control groups (Figure 1B). After 14 days, rats undergoing PWB40 were the only group performing below baseline values for front paw grip force, and significantly lower than all other groups (Figure 1C). In the hindlimbs (Figure 1D), a significant decline in grip force was evidenced as early as 7 days. RSV supplementation totally rescued grip force in both the front and hindlimbs of the rats exposed to partial unloading (Figures 1C,D).

**FIGURE 2:**
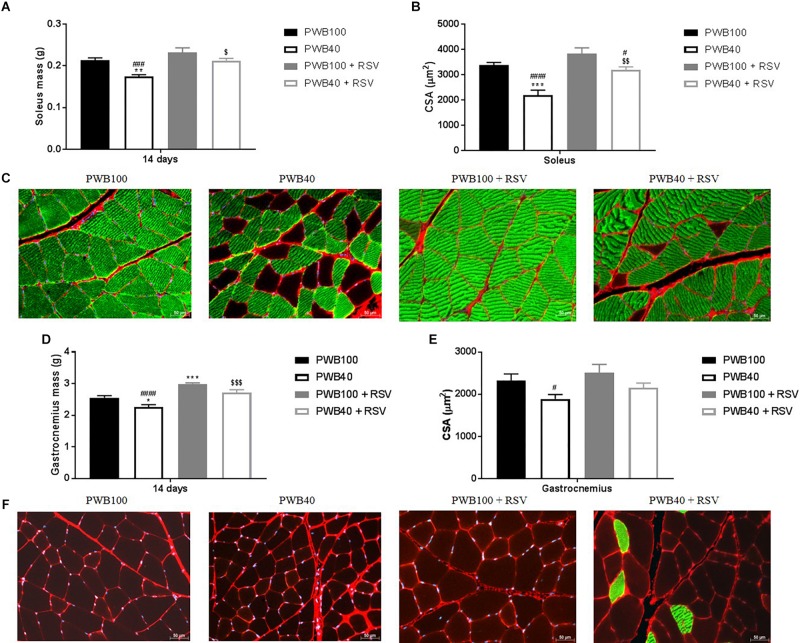
Terminal assessment of the muscles of the triceps surae, including soleus **(A)** and gastrocnemius wet mass **(D)**, soleus **(B)** and gastrocnemius **(E)** average cross sectional area (CSA), and representative images of the soleus **(C)** and gastrocnemius **(F)** muscles stained with anti-collagen VI (red), anti-slow-skeletal myosin heavy chain (green), and DAPI (blue). *N* = 6 per group. The results are presented as mean ± SEM; the results of the *post hoc* tests following the ANOVA are indicated. ^*^p < 0.05, ^∗∗^*p* < 0.01, and ^∗∗∗^*p* < 0.001 vs. PWB100, respectively. ^#^*p* < 0.05, ^###^*p* < 0.001, and ^#⁢#⁢#⁢#^*p* < 0.0001 vs. PWB100 + RSV, respectively. ^$^*p* < 0.05, ^$$^*p* < 0.01, and ^$$$^*p* < 0.001 vs. PWB40, respectively.

Data on terminal muscle assessment are presented in Figure 2 and Supplementary Tables S1, S2. Animals exposed to PWB40 alone displayed a significantly reduced soleus mass, average CSA, and slow-twitch-CSA compared to both non-unloaded groups. In contrast, RSV supplementation in animals exposed to PWB40 prevented muscle atrophy (Figure 2A) and partially rescued myofiber CSA (Figure 2B and Supplementary Table S2), but did not induce any significant change for the group at PWB100. As visualized in the images (Figure 2C), we observed a significant reduction of the myofiber type switch in the soleus of the rats undergoing PWB40 and supplemented with RSV (*p* = 0.0269 vs. PWB40, Supplementary Table S1). In the gastrocnemius, PWB40 induced a significant reduction of muscle mass (Figure 2D) and CSA (Figures 2E,F). RSV supplementation successfully increased muscle wet mass compared to non-treated animals (Figure 2D), but did not fully restore the average CSA (Figure 2E). However, the fiber-specific CSA did not significantly differ compared to the two non-unloaded groups (Supplementary Table S2). While a slight increase in slow-twitch myofibers was visible in the PWB40 + RSV group (Figure 2F), it was not significantly different compared to the other groups (Supplementary Table S1).

## Discussion

We previously demonstrated that our PWB system was well tolerated by the rats over a 14-day period (Mortreux et al., 2018, 2019) despite an initial body weight loss, similar to that observed in HLU models (Brooks et al., 2009) and analogous to what occurs in astronauts in flight (Matsumoto et al., 2011). As reported previously in healthy animals (Rauf et al., 2017), RSV did not affect food intake (Supplementary Table S3) or body weight (Figure 1A) in our study. In rodent models, grip force rapidly declines when animals are exposed to PWB (Wagner et al., 2010; Mortreux et al., 2018, 2019). Here we show that at all time-points, rats exposed to PWB40 + RSV did not score lower than their baseline values, or their non-unloaded counterparts. In both front and rear paw grip force experiments, the PWB40 cohort was the only group that displayed a significant decrease in grip force compared to their baseline (rear paw, Figure 1D) and that of the other groups (front paw, Figure 1C). As previously reported (Mortreux et al., 2018), we observed a slight recovery in rear paw grip force after 14 days of PWB40. The reason for this improvement is uncertain, but may be due, in part, to an adaptation to partial unloading or adult growth.

*Ex vivo* analysis highlighted the muscle-protective effect of RSV (Figure 2). In both the gastrocnemius and soleus muscles, we observed a significant rescue of muscle wet mass comparable to the one observed by Momken et al. (2011) after 14 days of HLU. These findings are further confirmed by the histomorphometric analyses. In the soleus, the average CSA in the PWB40 + RSV group was significantly higher than in the PWB40 group, but remained significantly lower than the PWB100 + RSV group. No differences were seen between the PWB40 + RSV group and the PWB100 controls. In the gastrocnemius, only the PWB40 group displayed a significantly lower average CSA compared to the PWB100 + RSV group. This could be due to the limited number of slides analyzed.

During spaceflight and in rodent models of mechanical unloading, a myofiber type switch has been described (Desplanches, 1997; Lomonosova et al., 2016; Shenkman, 2016; McDonald et al., 2017) in static, weight-bearing muscles such as the soleus, which predominantly consists of slow-twitch fibers, thus providing endurance over dynamic strength. Muscle deconditioning increases the proportion of type two fast twitch fibers in weight-bearing muscles, therefore making them prone to muscle fatigue. While our PWB40 animals displayed a significantly smaller percentage of type 1 fibers in the soleus compared to the normally loaded groups (Supplementary Table S1), rats undergoing PWB40 + RSV did not. This maintenance of muscle composition could explain the preservation of muscle function as demonstrated by our functional assessments. In contrast, gastrocnemius fiber type was not significantly altered by partial unloading or by RSV treatment, despite a 94.21% increase in type 1 myofiber compared to the PWB40 group.

Our initial functional study highlights the benefits of a moderate daily dose of RSV, however, several limitations are to be taken in consideration. First, our study involved only a modest number of male rats (*n* = 24), thus, we do not yet know if RSV supplementation would benefit females, similarly. Second, we assessed the effect of a single dosage of RSV (150 mg/kg/day). This choice was based on the literature and the known protective effects of RSV on bone (Durbin et al., 2014). Nevertheless, many dosages of RSV have been tested in animal models, up to 700 mg/kg/day (Williams et al., 2009), therefore, a more efficient dose to prevent muscle deconditioning during partial unloading could exist. Finally, our study assessed the functional outcomes of performance and composition of the main weight-bearing hindlimb muscles but did not focus on the deeper mechanisms and pathways that could be involved.

One possible explanation for our findings is that RSV could be responsible for maintaining skeletal muscle insulin sensitivity through its beneficial effect on glucose homeostasis and GLUT4 expression (Momken et al., 2011; Yonamine et al., 2017). Indeed, muscle is intrinsically linked to energy homeostasis since it is a major consumer of glucose during contraction. The transporter GLUT4 controls glucose uptake in the skeletal muscle, and when dysregulated, insulin resistance occurs (Dyar et al., 2014). This metabolic dysfunction has been observed in both astronauts and ground-based animal models of mechanical unloading (Tobin et al., 2002; Hughson et al., 2016; Gambara et al., 2017; Tascher et al., 2017). In diabetic animals, it has been shown that RSV treatment increases muscle weight and protein content by regulating GLUT4 expression in the skeletal muscle (Yonamine et al., 2017), while in healthy hypokinetic animals, RSV supplementation before, during, and after mechanical unloading, enhances insulin sensitivity and muscle recovery (Momken et al., 2011; Bennett et al., 2013), suggesting the presence of a significant link between glucose homeostasis and muscle health. Alternatively, this mitigating phenomenon could also be attributed to the anti-oxidative and anti-inflammatory effects of RSV, which could be crucial for the musculoskeletal system during a period of mechanical unloading, and are currently being investigated using other polyphenols such as dried plums (Simonavice et al., 2013; Schreurs et al., 2016). Further metabolic analyses will be required to unravel the cellular mechanisms involved in the muscle-protective effects of a moderate daily dose of RSV.

Taken together, our results highlight the therapeutic potential of RSV as a nutraceutical countermeasure to prevent muscle deconditioning in an animal model of Martian gravity. Further investigations should optimize the dose of RSV for the preservation of muscle function and explore the mechanisms involved. In addition, it will be important to confirm the lack of any potentially harmful interactions of RSV with other drugs administered to astronauts during space missions.

## Ethics Statement

All studies were approved by the Institutional Animal Care and Use Committee of Beth Israel Deaconess Medical Center and conform to the National Research Council “Guide for the Care and Use of Laboratory Animals.”

## Author Contributions

MM designed the methodology. MM and DR performed the experiments, and analyzed the data. MB and SR designed the study. All authors reviewed and approved the manuscript.

## Conflict of Interest Statement

The authors declare that the research was conducted in the absence of any commercial or financial relationships that could be construed as a potential conflict of interest.
