# Supplementary material for: A Moderate Daily Dose of Resveratrol Mitigates Muscle Deconditioning in a Martian Gravity Analog
Source: Front Physiol. 2019 Jul 18;10:899. doi: 10.3389/fphys.2019.00899 (PMC6656861; doi:10.3389/fphys.2019.00899)
Supplement: Supplementary file 1 [file Table_1.DOCX]

Supplemental Table 1. Percentage of slow-twitch myofibers in the triceps surae of the animals after 14 days. N=6/group. Results are presented as mean ± SEM and post hoc tests following one-way ANOVA are represented as *: p<0.05 vs PWB100, ##: p<0.01 vs PWB100+RSV, $: p<0.05 vs PWB40.

| %MyHC1 | PWB100 | PWB40 | PWB100+RSV | PWB40+RSV |
| --- | --- | --- | --- | --- |
| Soleus | 89.27 ± 3.89 | 77.18 ± 2.53^*##^ | 94.62 ± 2.87 | 90.13 ± 2.28^$^ |
| Gastrocnemius | 11.12 ± 1.78 | 9.32 ± 1.66 | 10.27 ± 4.30 | 18.1 ± 6.16 |

Supplemental Table 2. Fiber-specific cross sectional area (CSA) in the soleus and gastrocnemius muscles. N=6/group. Results are presented as mean ± SEM. The post hoc tests following the 1-way ANOVA are displayed as *: p<0.05 vs PWB100 and #, ##, ###: p<0.05, p<0.01, p<0.001 vs PWB100+RSV, respectively.

| Fiber-specific CSA | PWB100 | PWB40 | PWB100+RSV | PWB40+RSV |
| --- | --- | --- | --- | --- |
| Soleus MyHC1 | 3492 ± 105.8 | 2569 ± 283.7^*###^ | 3898 ± 202.2 | 3284 ± 128.8 |
| Soleus MyHC2 | 2674 ± 286.3 | 1959 ± 269.2 | 2630 ± 553.3 | 2309 ± 241.1 |
| Gastrocnemius MyHC1 | 1871 ± 170.2 | 1682 ± 290.3 | 1476 ± 461.1 | 1923 ± 245.6 |
| Gastrocnemius MyHC2 | 2394 ± 159.1 | 1912 ± 107.7^#^ | 2544 ± 176.7 | 2257 ± 120.8 |

Supplemental Table 3. Cumulative food intake per week. N=6/group. Results are presented as mean ± SEM. The post hoc tests following the ANOVA did not display any significant result.

| Cumulative food intake | PWB100 | PWB40 | PWB100+RSV | PWB40+RSV |
| --- | --- | --- | --- | --- |
| Week 1 | 163.17 ± 7.28 | 141.78 ± 12.73 | 170.77 ± 7.47 | 144.35 ± 9.85 |
| Week 2 | 167.85 ± 9.98 | 163.18 ± 15.02 | 196.18 ± 7.19 | 185.73 ± 6.11 |
